# Mapping Regional Changes in Multiple‐Timepoint Hyperpolarized Gas Ventilation Images and Validation by Radiologist Score

**DOI:** 10.1155/ijbi/1959442

**Published:** 2025-12-05

**Authors:** Ummul Afia Shammi, Talissa Altes, Cody Thornburgh, John P. Mugler, Craig H. Meyer, Kun Qing, X Eduard E. de Lange, Jaime Mata, Robert Thomen

**Affiliations:** ^1^ Richard and Loan Hill Department of Biomedical Engineering, University of Illinois Chicago, Chicago, Illinois, USA, uic.edu; ^2^ Radiology, School of Medicine, University of Missouri, Columbia, Missouri, USA, missouri.edu; ^3^ Radiology and Medical Imaging, University of Virginia School of Medicine, Charlottesville, Virginia, USA, virginia.edu; ^4^ Biomedical Engineering, University of Virginia, Charlottesville, Virginia, USA, virginia.edu; ^5^ Chemical and Biomedical Engineering, University of Missouri, Columbia, Missouri, USA, missouri.edu

## Abstract

**Background:**

Hyperpolarized gas (HPG) magnetic resonance imaging, recently FDA‐approved, offers an innovative approach to evaluating gas distribution and lung function in both adults and children.

**Purpose:**

In this study, we present an algorithm for calculating maps of changes in regional ventilation in asthma, cystic fibrosis, and COPD patients before and after receiving treatment. We validate the results with a radiologist′s evaluation for accuracy. Our hypothesis is that the change map would be in congruence with a radiologist′s visual examination.

**Assessment:**

Nine asthmatics, six cystic fibrosis patients, and five COPD patients underwent hyperpolarized 3He MRI. N4ITK bias correction, voxel smoothing, and normalization to the signal distribution′s 95th percentile voxel signal value were performed on images. For calculating regional ventilation change maps, posttreatment images were registered to baseline images, and difference maps were created. Difference‐map voxel values of > 60% of the baseline mean signal value were identified as improved, and those of < −60% were identified as worsened. In addition, short‐term improvement (STI) was identified where voxels improved at Timepoint 2 but returned to baseline at Timepoint 3. A grading rubric was developed for radiologist scoring that had the following assessment categories: “level of volume discrepancy” and “discrepancy causes” for each ventilation change map.

**Results:**

In 15 out of the 20 cases (75% of the data), there was a small to no volume disparity between the change map and the radiologists′ visual evaluation. The rest of the two cases had moderate volume differences, and three cases had large ones.

**Conclusion:**

Our regional change maps demonstrated congruence with visual examination and may be a useful tool for clinicians evaluating ventilation changes longitudinally.

## 1. Introduction

Hyperpolarized gas (HPG) magnetic resonance imaging (MRI) is an innovative imaging technique that can play a vital role in assessing gas distribution and lung function in adults and children [1, 2], which has recently received FDA approval for clinical use [3]. HPG imaging yields high‐quality images showing the physiologic spatial distribution of an inhaled bolus of inert gas inside the lung, which allows for the localization of problematic areas due to lung disease. A large number of drug development studies have been conducted with HPG MRI, which can make an impact on clinical management by enabling functional response to lung therapy [4–8]. From a clinical perspective, its use in the characterization of regional lung function in difficult lung diseases is the likely area of immediate clinical impact.

There are several methods that have been established to quantify the total ventilation defect percentage (VDP) of the lung before and after the drug with HPG MRI. Regions of low signal in HPG images are identified and quantified as a subject′s VDP [9–17]. Although VDP has been shown to be more sensitive than spirometry at quantifying lung function [17], it is a global measure of lung function, and information related to regional function is absent. Previously, a method was established for mapping quantitative treatment response to therapy, but its performance has not been benchmarked against a radiologist′s score [18]. There is a need for analyses that can quantify regional changes in lung ventilation within a single patient that are validated by radiologist inspection, as this would allow for assessment of longitudinal treatment efficacy and/or disease progression across multiple timepoints.

In this study, we propose a method for quantifying regional ventilation changes with multiple‐timepoint HPG MRI in one set of images and quantify the total improvement/worsening of the lung following the treatment. The regional ventilation change maps were observed and scored by two expert radiologists in a blinded fashion. Our objective is to visualize and compute regional ventilation change maps from HPG images so that they reflect the visual changes in lung ventilation in patients with asthma, COPD, and cystic fibrosis following the drug treatment. As emerging therapies are geared toward preventing or delaying permanent lung pathologies, we hope that measuring such local changes pre–/post–drug treatment would be beneficial for clinicians′ evaluation of treatment response. We hypothesized that quantitative change maps would mimic radiologists′ interpretation in determining regional ventilation changes in hyperpolarized ^3^He MRI of asthma subjects following bronchodilator.

## 2. Methods and Materials

### 2.1. Subjects and Protocol

We present an analysis of MR data obtained from several distinct studies. These studies were approved by the Institutional Review Board, and all subjects gave informed, written consent prior to study enrollment. Nine asthmatics (age 24.5 ± 5.9), six cystic fibrosis patients (age 18.8 ± 4.6), and five COPD patients (age 61 ± 7.9) were recruited for separate studies. All subjects provided written informed consent. Patient demographics and pulmonary function test (PFT) results are summarized in Table 1. The asthma study was performed on a 1.5‐T whole‐body MR scanner (MAGNETOM Avanto; Siemens, Malvern, Pennsylvania) that was modified with a broadband amplifier to allow operation at the ^3^He resonant frequency. Each subject underwent HP helium‐3 MRI and spirometry on the same day at three different timepoints: (1) at baseline, (2) 30–60 min after receiving the bronchodilator, and (3) 4–5 h after receiving the bronchodilator (Figure 1). A short‐acting beta agonist (SABA‐albuterol) was administered via inhaler.

**Table 1:** Demographic description of subjects with CF and COPD.

**Parameter**	**Asthma**	**CF**	**COPD**
Age	24.5 ± 5.9	18.8 ± 4.6	61 ± 7.9
Gender	4M/5F	4M/4F	3M/2F
Height (m)	1.6 ± 0.12	1.6 ± 0.1	1.6 ± 0.05
Weight (lb)	69.8 ± 12	66.3 ± 14	64.4 ± 9
BMI	24.82 ± 3.38	23.52 ± 3.5	23.34 ± 3.5
FEVpp	69 ± 19.23	95.3 ± 22.2	49.7 ± 9.7
FVCpp	89.4 ± 8.4	94.2 ± 14.6	65 ± 13.40

Figure 1Study design for (a) asthma, (b) cystic fibrosis, and (c) COPD study.

**Figure:**
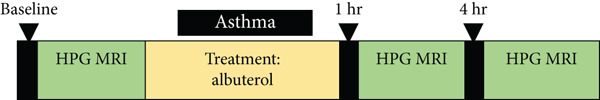
(a)

**Figure:**
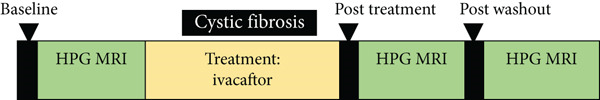
(b)

**Figure:**
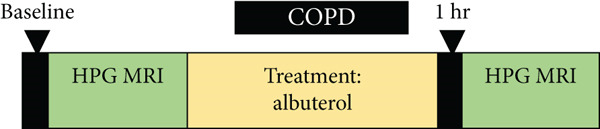
(c)

For the CF study, patients aged 12 years or older with CF were eligible for study inclusion. They are required to have at least one G551D mutation and ppFEV1 (percent predicted FEV1) of at least 40 at screening. Subjects underwent HP ^3^He gas MRI following administration of ivacaftor [19], which has been shown to improve lung function, reduce pulmonary exacerbations, and improve symptoms of CF in eligible patients. A 3D balanced steady‐state free precession (b‐SSFP) sequence was used for acquiring ^3^He HPG MRI and proton images on a 1.5 T Siemens Avanto scanner at the same breath hold. Images were analyzed at three timepoints: baseline, posttreatment (4 weeks after baseline), and postwashout (6 weeks after baseline) (Figure 1).

For the COPD study, scanning was performed in a 1.5 T MR scanner using a gradient echo (GRE)–based pulse sequence. Each subject was given Helium‐3 and inhaled albuterol in between the same‐day imaging sessions (Figure 1). The images pre‐ and postalbuterol were quantitatively analyzed. The imaging parameters for the three studies are listed in Table 2 (total readout bandwidth equals pixel bandwidth multiplied by the number of frequency‐encoding points).

**Table 2:** Summary of MR pulse sequence acquisition parameters.

**Metric**	**Asthma**	**CF**	**COPD**
Acquisition matrix	256 × 256/128 × 80	128 × 80	128 × 80
Orientation	Coronal/axial	—	Axial
Sequence	2D FLASH	3DTrueFISP	2D FLASH
# of slices	~34	52	23
Slice thickness	10 mm	3.9 mm	15
Pixel size (mm)	3.28 × 3.28	3.9 × 3.9	3.125 × 3.125
Flip angle (°)	9°	9°	10°
TR/TE (msec)	7/2.7	1.86/0.79	6.7/2.85
F0V	42 × 26.2 cm^2^	50 × 31 cm^2^	40 × 25 cm^2^
Acquisition time (s)	~12	~12	~12
Pixel BW (Hz/Px)	200	1085	200

### 2.2. Image Analysis

Manual segmentation of the lung from proton breath‐hold images was performed separately using 3D slicer image analysis software [20], and descriptive analyses of the images were performed using custom software in the R statistical computing platform. N4ITK bias correction [21] and voxel smoothing were performed on images, which were normalized to the signal distribution′s 95th percentile voxel signal value. Ventilation defects were identified in each image set by calculating the percentage of lung voxels with less than 60% of the mean whole‐lung HP gas signal. A median filter (3 × 3 kernel) was then applied to the resulting binary defect map [9] to remove defects smaller than five voxels (considered inconsequential) and to smooth the defect contours. The VDP was calculated as the percentage of the whole‐lung volume marked as a defect. Signal‐to‐noise ratios (SNRs) were calculated for each acquired image set. The SNR was determined by computing the signal within the lung region and a manually chosen area outside the lung for noise characterization:

SNR=meansignal−meannoisesdnoise,

where sd(noise) mean the standard deviation of the noise profile.

**Figure 2:**
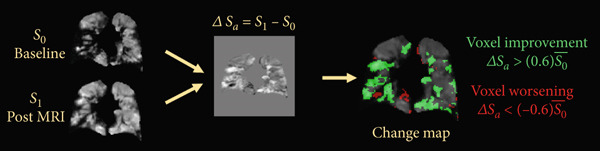
Illustration of ventilation improvement/worsening analyses. The regional changes were only calculated within lung voxels segmented from proton images. Regions of improvement/worsening in the ventilation pattern are denoted in green/red color.

**Figure 3:**
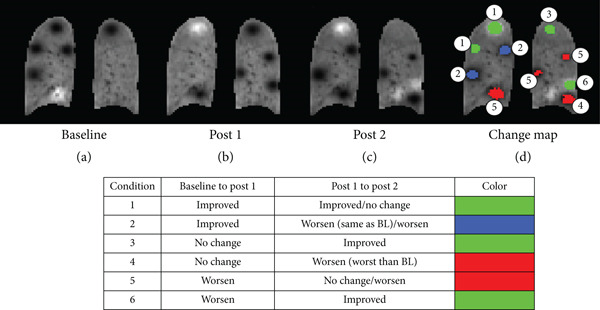
Illustration of ventilation improvement/worsening analyses for a three‐timepoint analysis. (a) Baseline, (b) Post 1, (c) Post 2, and their corresponding (d) change map. Defects were synthesized using a Gaussian function for 10 different cases highlighted in the table. The regional changes were only calculated within lung voxels segmented from proton images. Regions of improvement/short‐term improvement/worsening in the ventilation pattern are denoted in green/blue/red color. BL, baseline; STI, short‐term improvement.

For calculating regional ventilation change maps, each subject′s baseline images were registered to corresponding posttreatment images by applying a nonrigid affine transformation using custom software in Matlab (MathWorks, Natick, Massachusetts). The difference in the voxel signal intensity was calculated for baseline (*S*_0_) to posttreatment (*S*_1_)(*Δ* *S* = *S*_1_ − *S*_0_) (Figure 2). Difference‐map voxel values of > 60% of the baseline mean signal value were identified as improved, and those of < −60% were identified as worsened. Figure 2 shows a change map where the improvement and worsening of the lung posttreatment are shown in green and red colors, respectively. For the asthma and CF study, which involved three imaging timepoints, three change maps were created: baseline to Post 1 (*Δ* *S*_*a*_ =*S*_1_ − *S*_0_), Post 1 to Post 2 (*Δ* *S*_*b*_ =*S*_2_ − *S*_1_), and baseline to Post2 (*Δ* *S*_*c*_ =*S*_2_ − *S*_0_). A voxel was classified as a “sustained improved region” if the signal in that region increased from baseline to Post 1 (meeting the condition *Δ* *S*_*a*_ > 0.6∗*S*_0_) and remained elevated from Post 1 to Post 2 (meeting *Δ* *S*_*b*_ > 0.6∗*S*_1_). These regions are shown in green on the change map. This definition was chosen to emphasize persistence of improvement across consecutive timepoints, rather than only relative to baseline. Similarly, a voxel was classified as a “worsening region” if the signal in that region decreased from baseline to Post 1 (meeting *Δ* *S*_*a*_ < 0.6∗*S*_0_) and continued to decrease from Post 1 to Post 2 (meeting *Δ* *S*_*b*_ > 0.6∗*S*_1_). These regions are indicated in red. Finally, a voxel was classified as a “short‐term improvement region” if the signal increased from baseline to Post 1 (meeting *Δ* *S*_*a*_ > 0.6∗*S*_0_) but then decreased from Post 1 to Post 2 (meeting *Δ* *S*_*b*_ < 0.6∗*S*_1_). These regions are marked in blue on the change map. Similarly, there are 10 possible physiological conditions that can arise from the three imaging timepoints. These cases, along with the color‐coding criteria, are highlighted in Figure 3. Defects were synthesized using a Gaussian function with different radii for creating all the possible cases of improvement/worsening in three sessions. Change maps were superimposed on Post 2 images in an effort to simulate radiologists′ evaluation of changes in regional ventilation after treatment. Finally, ventilation changes from baseline to posttreatment were expressed as a percentage of whole‐lung volume and defined as regional percent change (RPC), a collective term representing three regional measures: sustained improvement, short‐term improvement, and worsening.

The change maps were reviewed by two radiologists experienced in HPG MRI: one with 25+ years of experience and the other with 3 years. Both received standard training and guidance for analyzing defects in HPG MR images. Each case was presented on its own sheet per subject. The images were assessed by each radiologist independently and then cooperatively to achieve consensus; radiologists were blinded to the subject disease and medication status. They analyzed their agreement with the reported quantitative ventilation change maps for two‐/three‐timepoint studies (Figure 4). A grading rubric was developed to score each ventilation change map based on the “level of volume discrepancy” (Figure 4). This volume discrepancy refers to any differences observed between the overall volume of the change map and the radiologist′s visual assessment. These discrepancies were categorized into four levels: large, moderate, small, or none. A “none” score indicates that the algorithm′s output perfectly matches the radiologist′s observations. In contrast, a “large” score means that there is a significant difference between the algorithm′s detected changes and the radiologist′s visual interpretation, indicating potential issues with the algorithm′s detection of ventilation changes. Additionally, the radiologists identified the causes of the discrepancies, whether due to under‐ or overdetection by the algorithm or due to inaccuracies in registration or segmentation during preprocessing. No image processing was performed on the hyperpolarized images displayed to the radiologists for evaluation.

Figure 4(a) Example ventilation images (Subject 8 without preprocessing) and the corresponding regional change maps. (b) Scoring sheet that was presented to two expert radiologists. Green: improvement; blue: short‐term improvement (STI); red: worsening.

**Figure:**
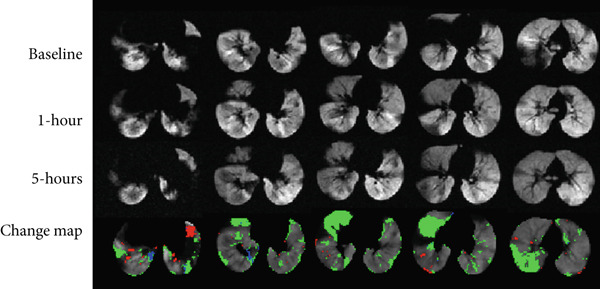
(a)

**Figure:**
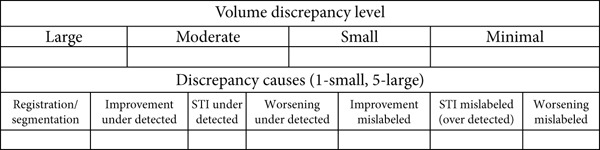
(b)

### 2.3. Statistical Analysis

Box plots were created for RPC to find the total change in lung volume posttreatment. Pearson′s correlation coefficient was calculated between RPC, VDP, and FEV1 to measure the relation between these quantitative metrics. *p* values less than 0.05 were considered significant. Line plots were made to demonstrate the VDP change after the treatment in three populations.

## 3. Results

HPG MRI procedures were well tolerated by all subjects, and no serious or severe adverse events were reported. The average VDP at baseline, 1 h, and 5 h was 15*%* ± 8*%*, 9.7*%* ± 6.7*%*, and 12*%* ± 7.5*%*, respectively. On average, nine asthmatic subjects showed 0.72*%* ± 7.54*%* sustained improvement, 0.98*%* ± 1.67*%* short‐term improvement, and 1.18*%* ± 0.90*%* worsening in lung ventilation (Figure 5). More than 5% sustained improvement in the lung was shown for Asthma Subjects 2, 5, 6, 7, and 8 among all nine cases. The average SNR was 35 ± 13.

Figure 5Subject response to treatment is quantified with ventilation defect percentage (VDP) shown in (a)–(c) for asthma, cystic fibrosis, and COPD, respectively. Box plots of lung volume percentage that experiences improvement, STI, or worsening for all subjects shown in (d)–(f).

**Figure:**
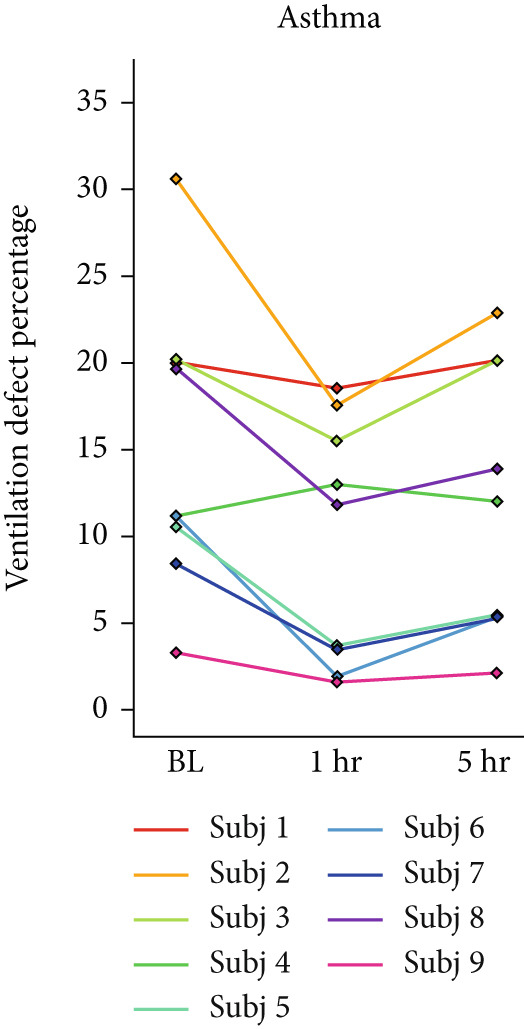
(a)

**Figure:**
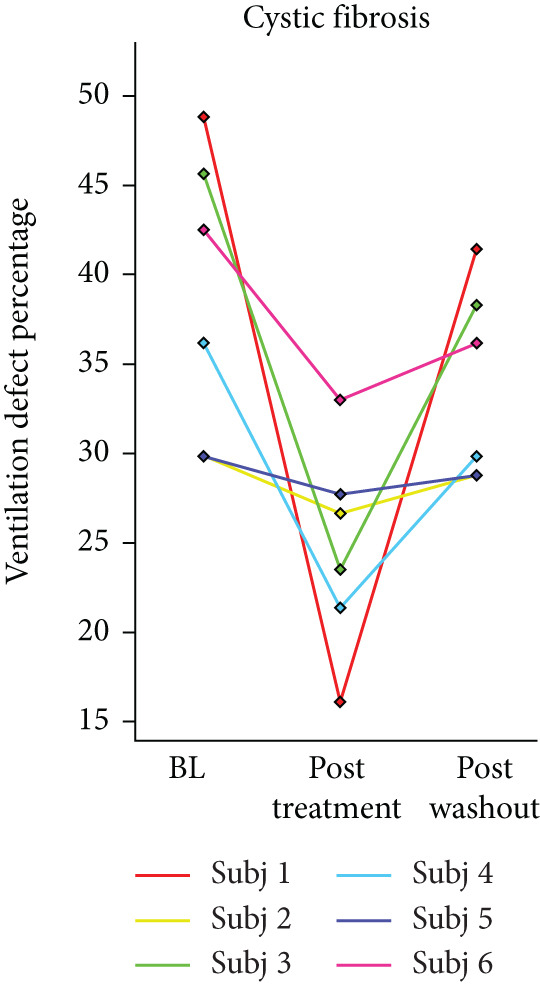
(b)

**Figure:**
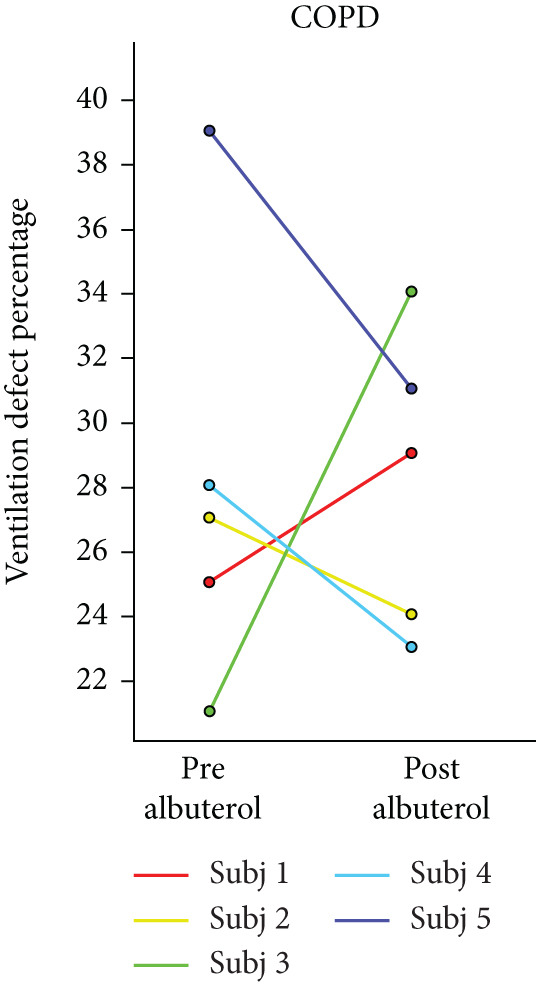
(c)

**Figure:**
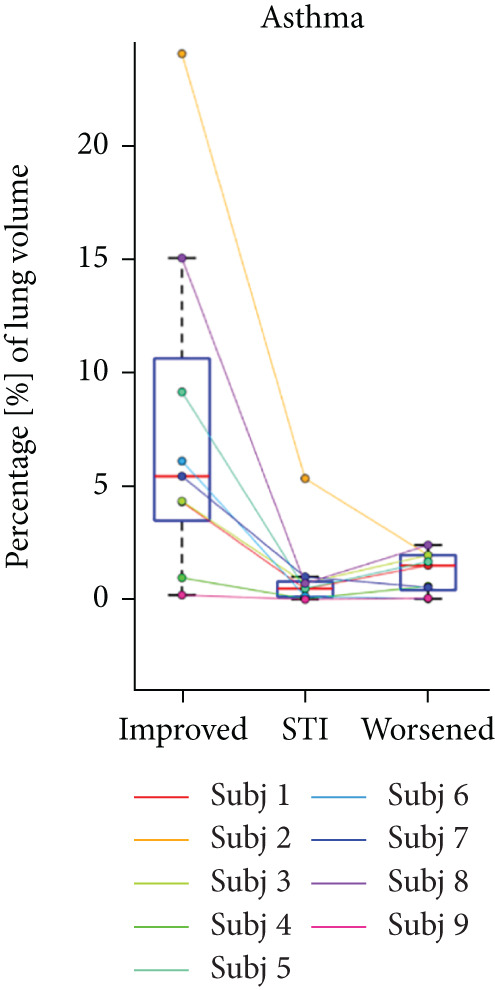
(d)

**Figure:**
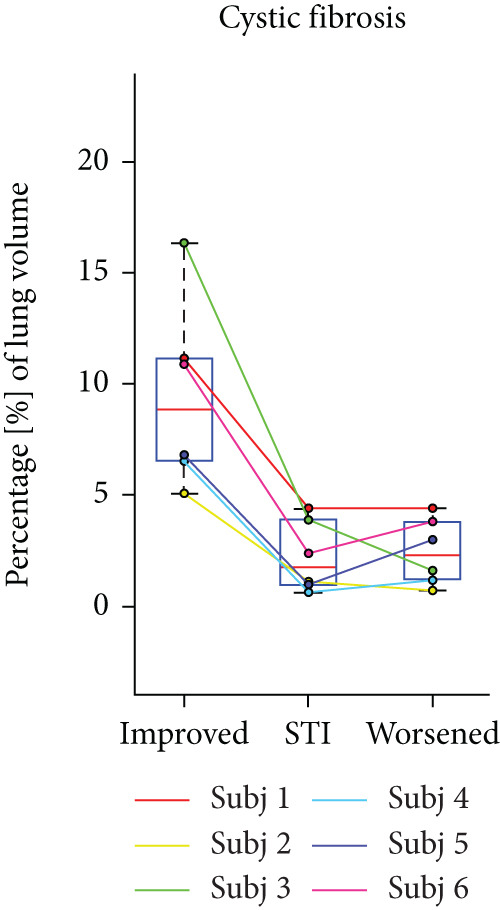
(e)

**Figure:**
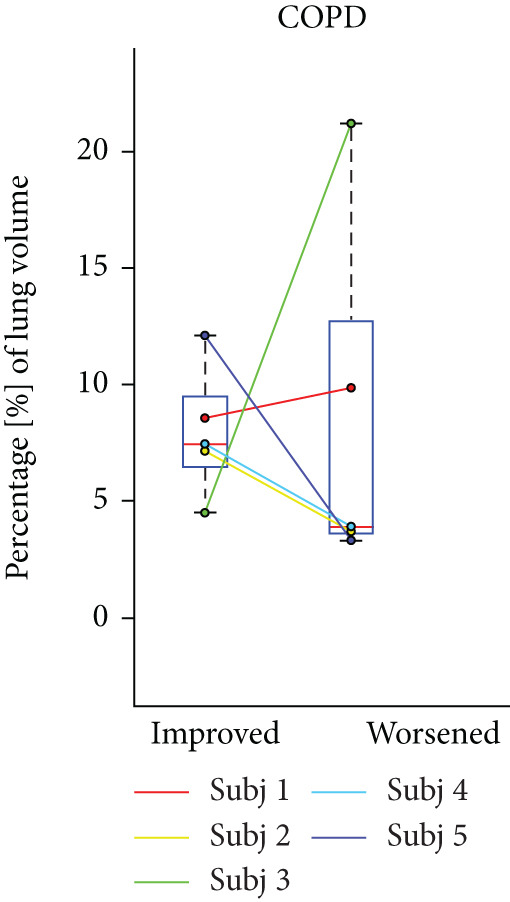
(f)

For the CF study, the average VDP at baseline, posttreatment, and postwashout was 38.5*%* ± 7.7*%*, 25.2*%* ± 5.5*%*, and 33.8*%* ± 5.2*%*, respectively. The calculated RPC indicates that there was 9.5*%* ± 4.16*%* sustained improvement, 2.3 ± 1.6 STI, and 2.5 ± 1.5 worsening postwashout (Figure 5). The average SNR, including all sessions, was 63 ± 23.

The average VDP for COPD subjects at baseline and posttreatment was 28*%* ± 6.7*%* and 28*%* ± 4.6*%*, respectively. On average, there was 7.9*%* ± 2.7*%* improvement and 8.34*%* ± 7.6*%* worsening of postalbuterol (calculation from the first imaging session for five subjects). More than 5% sustained improvement in the lung was shown in four of the five cases (Figure 5). The average SNR for all sessions was 26.2 ± 1.

For the asthma and CF study, the correlation between the sustained improvement and *Δ*VDP of baseline and after two sessions was 0.78 (*p* < 0.005). The correlation between the short‐term improvement and *Δ*VDP of baseline and after one session was 0.74 (*p* < 0.005).

**Figure 6:**
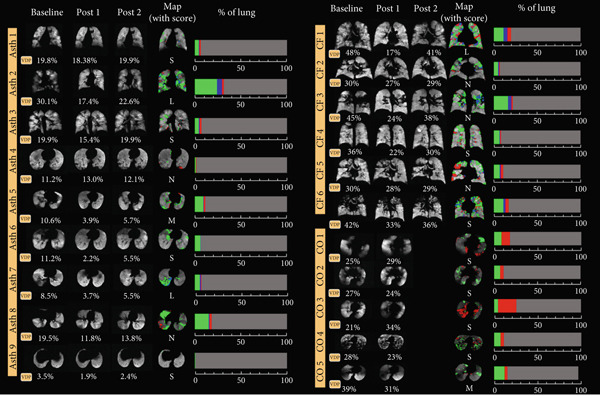
Representative slices for all subjects alongside whole‐lung VDP measurement, change in percentage (%) of lung, and radiologist‐scored volume discrepancy for each scan. Asth: asthma; CF: cystic fibrosis; CO: COPD; VDP: ventilation defect percentage; S: small; M: medium; L: large; N: none.

Figure 6 shows a representative slice for each subject at two/three imaging sessions and the corresponding score given by the radiologist. In total, radiologists scored 20 cases in total. In 15 out of the 20 cases (75% of the data), there was a small to no volume disparity between the change map and the radiologists′ visual evaluation. The rest of the two cases had moderate volume differences, and three cases had large ones. The change maps for Asthma Subjects 2 (VDP baseline 30%) and 7 (VDP baseline 8.5%), as well as CF Subject 1 (VDP baseline 48%), were scored with large volume discrepancies (Figure 7). The average VDP at baseline was 28 ± 19 for three cases that exhibited “large” volume disparities. The radiologist reported that a significant volume disparity for Asthma Subject 7 was caused by incorrect slice registration. In addition, STI was mislabeled as an improvement in Asthma Subject 2 and CF Subject 1.

Figure 7Radiologists scored the volume discrepancy level as large in (a) Asthma Subject 2 and (b) Asthma Subject 7. Areas of short‐term improvement were erroneously identified as improved regions in Asthma Subject 2. Asthma Subject 7 had misregistered slices above the carina.

**Figure:**
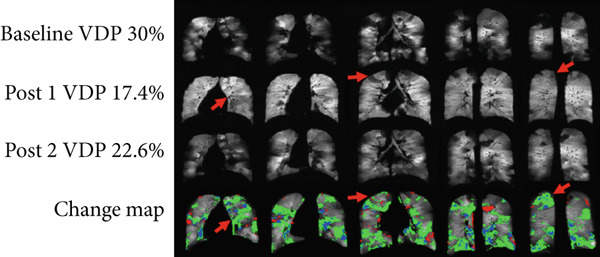
(a)

**Figure:**
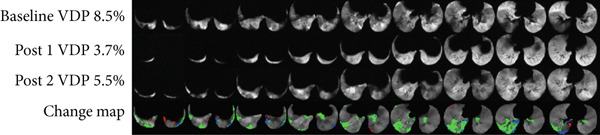
(b)

## 4. Discussion

Our regional change maps showed congruence with visual assessment across different populations and could be a helpful tool for physicians analyzing ventilation changes over time. Spirometry, gas transfer test, or static lung volume measurements are frequently used in clinical evaluations for treatment efficacy. Although these techniques are quick and simple to repeat, they cannot be used to assess regional variations in lung ventilation—which may be useful in studies of targeted treatments. In particular, if medical treatment exclusively targets a specific region of the lung, then the conventional clinical tests that are considered to be the gold standard may not provide the entire picture. HPG MRI as a diagnostic tool can provide regional insight into changes in both the structure and the function of the lung. To our knowledge, this is the first time that the regional ventilation change map of HPG MRI has been scored by an expert over multiple timepoints and exhibited good agreement.

While we included available FEV1 data at each timepoint for completeness (Table 3), the correlation between *Δ*FEV1 and the imaging‐derived RPC was weak (*R* ^2^ = 0.10 for all subjects). This was not unexpected. FEV1 represents a global, bulk measure of airway obstruction, whereas HPG MRI provides regional maps of ventilation heterogeneity. Subtle or localized changes—especially those involving small airway recruitment or transient bronchodilator effects—may not produce measurable differences in whole‐lung spirometric indices. Therefore, the lack of correlation between *Δ*FEV1 and imaging‐derived metrics reflects the fundamental difference in what these measures capture: FEV1 represents a global index of airflow limitation, whereas HPG MRI quantifies spatially heterogeneous ventilation. Thus, the imaging‐derived regional changes provide complementary information beyond what can be inferred from conventional spirometry.

**Table 3:** VDP and FEV1% predicted values across timepoints.

**Asthma**						
**Subject**	**Baseline**		**1 h**		**5 h**	
	**VDP**	**ppFEV1**	**VDP**	**ppFEV1**	**VDP**	**FEv1pp**

1	19.82	86	18.38	97	19.94	78
2	30.07	39.2	17.44	90.53	22.6	72
3	19.99	44	15.41	48	19.96	43
4	11.25	95.6	13.02	117	12.02	128
5	10.62	68.93	3.98	88	5.72	61
6	11.2	88.35	2.25	113	5.59	107
7	8.56	72.67	3.77	96	5.49	78
8	19.51	63.76	11.86	74	13.89	70
9	3.57	63.11	1.94	81	2.44	76

**CF**						
**Subject**	**Baseline**		**Posttreatment**		**Postwashout**	
	**VDP**	**ppFEV1**	**VDP**	**ppFEV1**	**VDP**	**ppFEV1**

1	48	62	17	83	41	72
2	30	92	27	95	29	89
3	45	58	24	89	38	64
4	36	111	22	113	30	108
5	30	124	28	134	29	126
6	42	92	33	107	36	96

**COPD**						
**Subject**	**Pre**			**Post**		
	**VDP**	**ppFEV1**		**VDP**	**ppFEV1**	

1	25	37.99		29	52.28	
2	27	37.67		24	51.80	
3	21	65.04		34	61.34	
4	28	60.31		23	56.63	
5	39	43.45		31	N/A	

Abbreviations: N/A, data not available; ppFEV1, percent predicted forced expiratory volume in 1 s; VDP, ventilation defect percentage.

The algorithm was successful in detecting the overall improvement in the majority of the cases (75% of the cases), but it occasionally had trouble detecting STIs that were noticed by radiologists. They pointed out that the identified inconsistencies in the change maps can be significantly influenced by misregistration and inaccurate segmentation (Asthma Subject 7). Coil sensitivity may also play a role since the radiologist compared original pre‐/postimages without any bias correction. Nevertheless, the accuracy of coregistration was thoroughly evaluated through visual inspection, ensuring that the images were properly aligned without significant deformation.

In longitudinal inquiry and therapy evaluation in HPG MRI investigations, VDP has drawn a lot of interest [9]. Our algorithm shows a strong correlation (*ρ* = 0.78 and *p* < 0.005) between sustained improvement after three sessions and change in VDP (before vs. after two sessions).

The specific case—Asthma Subject 2 (Figure 7), where the radiologist scored volume discrepancies as large—was a difficult case since the subject had many defects that resolved 1 h following albuterol but returned after 5 h. The change map detected the improvement successfully but struggled to detect the STI. This is likely due to the use of a strict threshold across all cases—a known limitation of histogram‐based VDP algorithms [21]. We tested our algorithm with different threshold values (0.2–0.8) and observed that lowering the threshold finds more short‐term improvement regions. Nevertheless, in the majority of cases, the fixed threshold (0.6) that we used supported radiologist consensus and may offer a helpful indicator of regional ventilation changes to evaluate the time course of treatment effectiveness. Unlike rigid threshold‐based approaches, radiologist reads are less prone to nonphysiologic sources of signal variance, including coil sensitivity, low SNR, and variable lung inflating volumes. Incorporating radiologist input in future studies may allow for quicker refinement of techniques for clinical translation and may better reflect endpoints used in clinical practice, separate from research.

Our method extends prior treatment–response mapping [18] approaches by incorporating multitimepoint analysis and direct radiologist validation, providing temporally resolved, clinically interpretable ventilation change maps that remain consistent with established ^3^He/^129^Xe quantification methods. In this study, we hypothesized that regions of overall improvement/worsening/short‐term improvement could be delineated; however, this technique could be extended to identify other longitudinal ventilation changes of interest. We chose to use a three‐color map to identify the activation timepoints for a particular medicine, but the colors can be altered for various categories, such as short‐term worsening and upward/downward trending ventilation. Additionally, the algorithm performs effectively across varying slice thicknesses and different sequences in ventilation images. The mean SNR across all subjects was 42.6 ± 22. The SNR did not impact VDP calculation, as VDP is consistent for SNR values above approximately 10 [12]. The scan‐to‐scan variability was minimal since data acquisition was conducted under consistent protocols, ensuring uniformity across scans.

Limitations of this study included the relatively small sample size. We also recognize the potential for error in the manual segmentation/registration of various populations because proton images were not collected during the same breath hold. This may introduce errors in measurements, as regions that overlap imperfectly may yield large discrepancies in reported ventilation changes. Radiologist assessment of the data and analyses is important to identify these discrepancies. Careful consideration was given while performing manual segmentation and registration. Use of the same breath‐hold anatomic images in segmentation/registration may improve accuracy/repeatability, though radiologist assessments and a low number of major discrepancies due to registration/segmentation in the presented results are encouraging. This algorithm has been tested on HPG ^3^He images only, but it is expected to work similarly on HPG ^129^Xe images since both are spin density–based, and the 60% threshold for VDP calculation has also been effective with HPG ^129^Xe [9]. In the future, we plan to create an automated software for calculating regional change maps with a user interface that considers various thresholds. This may be a useful aid for clinicians in order to assess regional treatment response or guide targeted therapies in a single image set.

## 5. Conclusion

We presented a method to calculate regional ventilation change maps to quantify the temporal effects of drug treatment in asthma, cystic fibrosis, and COPD subjects. In the future, we intend to apply this method to a wider range of lung conditions and larger datasets. This could be crucial for determining how well‐targeted medicines work.

## Conflicts of Interest

X Eduard E. de Lange reported financial support from the National Institutes of Health (NIH), National Heart, Lung, and Blood Institute (NHLBI) under Grant No. R01 HL66479. Talissa Altes reports financial support from Vertex Pharmaceuticals Incorporated, as well as from GlaxoSmithKline, alongside John Mugler III. Talissa Altes also reports a consulting or advisory relationship with Vertex Pharmaceuticals. All other authors declare that they have no known competing financial interests or personal relationships that could have appeared to influence the work reported in this paper.

## Funding

This study was funded by the National Heart, Lung, and Blood Institute, 10.13039/100000050, R01 HL66479; the Vertex Pharmaceuticals, 10.13039/100011022; the GlaxoSmithKline foundation, 10.13039/501100002066.

## Data Availability

Data subject to third‐party restrictions (for Vertex Pharmaceuticals data).
